# Small-bowel necrosis complicating a cytomegalovirus-induced superior mesenteric vein thrombosis in an immunocompetent patient: a case report

**DOI:** 10.1186/1752-1947-6-118

**Published:** 2012-04-24

**Authors:** John Kalaitzis, Paris Basioukas, Eftalia Karzi, Charalampos Markakis, Emmanouil Liarmakopoulos, Andreas Hadjimarkou, Spyros Rizos

**Affiliations:** 1 First Surgical Department, Tzaneio General Hospital, Piraeus, Greece; 2 Radiology Department, Tzaneio General Hospital, Piraeus, Greece

**Keywords:** CMV, superior mesenteric vein, thrombosis

## Abstract

**Introduction:**

Superior mesenteric venous thrombosis as a result of acute cytomegalovirus infection is rare, with only a few cases reported in the literature.

**Case presentation:**

We present the case of a 40-year-old Caucasian man who was admitted to our hospital with a 5-day history of fever. His serological test and pp65 antigen detection of cytomegalovirus were positive, suggesting acute infection. On the sixth day after his admission, the patient complained of acute, progressive abdominal pain. Abdominal computed tomography revealed acute superior mesenteric venous thrombosis. An emergency laparotomy showed diffuse edema and ischemic lesions of the small bowel and its associated mesentery with a 50-cm-long segmental infarction of the proximal jejunum. An extensive enterectomy of about 100 cm of jejunum that included the necrotic segment was performed, followed by an end-to-end anastomosis. Anti-coagulation therapy was administered pre-operatively in the form of small-fractionated heparin and continued postoperatively. The patient had an uneventful recovery and was discharged on the 11th postoperative day.

**Conclusion:**

Acute cytomegalovirus infection can contribute to the occurrence of mesenteric venous thrombosis in immunocompetent patients. It is important for physicians and internists to be aware of the possible thrombotic complications of cytomegalovirus infection. A high level of clinical suspicion is essential to successfully treat a potentially lethal condition such as superior mesenteric venous thrombosis.

## Introduction

Mesenteric venous thrombosis accounts for 5% to 15% of all mesenteric ischemic events and usually involves the superior mesenteric vein [1]. However, superior mesenteric venous thrombosis as a result of acute cytomegalovirus (CMV) infection is extremely rare, especially in immunocompetent patients [2]. We present the case of an immunocompetent man with acute superior mesenteric vein thrombosis and small-bowel infarction as a result of CMV infection.

## Case presentation

A 40-year-old Caucasian man with a body mass index (BMI) of 29 was admitted to our hospital with a 5-day history of fever. His physical examination revealed anorexia and fever (temperature > 39°C). His white blood cell count was 11,350 cells/mm^3 ^with 5800 lymphocytes/mm^3^. His C-reactive protein level was 51 mg/L. His liver function test results were alanine aminotransferase 68 IU/L and aspartate aminotransferase 75 IU/L. His prothrombin and partial thromboplastin times were within normal limits. Multiple blood cultures were negative.

The results of serological tests for human immunodeficiency virus (HIV) enzyme-linked immunosorbent assay (ELISA), hepatitis A immunoglobulin M (IgM), hepatitis B surface antigen, hepatitis C virus, Coxsackie B virus and toxoplasmosis were negative. Viral capsid antigen and Epstein-Barr nuclear antigen IgG antibodies were positive, suggesting a past infection with Epstein-Barr virus. The serological test for CMV ELISA was positive for IgM antibodies (CMV IgM > 50 U/ml). The result of a CMV pp65 antigenemia assay was also positive, suggesting acute CMV infection.

**Figure 1:**
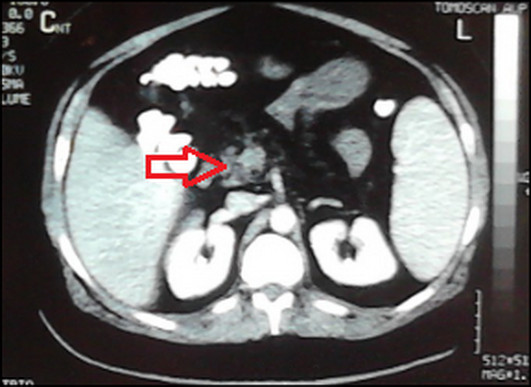
**Abdominal computed tomographic scan showing the presence of a thrombus about 7 mm in diameter at the proximal superior mesenteric vein**.

On day 6 after admission, the patient complained of diffuse abdominal pain. An abdominal ultrasound revealed a small increase in the size of the spleen and the presence of free peritoneal fluid. During the next 24 hours, his abdominal pain worsened with the addition of rebound tenderness. Abdominal contrast-enhanced computed tomography revealed the presence of a thrombus about 7 mm in diameter located at the proximal superior mesenteric vein (Figure 1).

**Figure 2:**
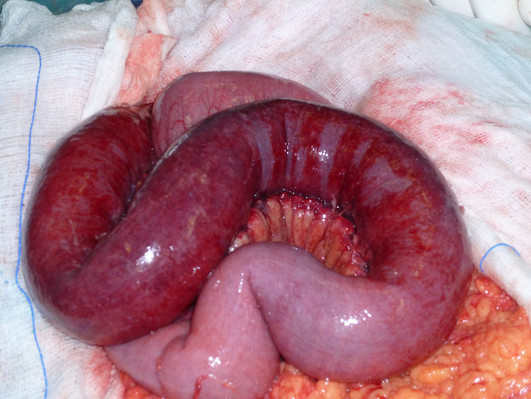
**Photograph showing the small bowel and its associated mesentery with diffuse edema and ischemic lesions**. A segmental infarction of the jejunum is present.

An emergency laparotomy was performed. The small bowel as far as the distal ileum and its associated mesentery had diffuse edema and ischemic lesions (Figure 2). Furthermore, a segmental infarction of the proximal jejunum 50 cm long was present. An extensive enterectomy of about 100 cm of jejunum, including the necrotic segment, was performed, followed by an end-to-end anastomosis.

Neither the patient nor his first-degree relatives had a history of vasculopathy or thrombosis. Anti-coagulation therapy was administered preoperatively when the diagnosis was established in the form of small-fractionated heparin (enoxaparin sodium 6000 IU twice daily) and continued for 1 month postoperatively. The patient had an uneventful recovery and was discharged on the 11th postoperative day.

One month later the patient underwent a complete coagulation profile check, including anti-phospholipid antibody, factor VIII, protein C and protein S levels, as well as factor V Leiden and prothrombin 20210A mutations, that showed no deficiency. Six months later the patient was healthy and free of any symptoms.

## Discussion

An increasing body of evidence suggests a causal association between CMV infection and vascular complications. CMV deoxyribonucleic acid (DNA) has been found in venous and arterial walls, which present a site of latency for this virus [3]. Vascular infection and consequent inflammation is thought to produce occlusive vascular ischemia, especially in patients with acquired immune deficiency syndrome (AIDS) or in those who have undergone transplantation and high-dose immunosuppressive therapy [4,5].

However, CMV-induced venous thrombosis in immunocompetent patients with no known coagulopathy is rare, with only a few cases reported in the literature [6]. In a review by Abgueguen *et al*., 11 patients were identified, comprising 8 women and 3 men, all of whom were under 50 years of age [7]. Seven patients had portal and/or superior mesenteric venous thrombosis. Most patients showed no predisposing factors for thrombosis.

The exact mechanism of CMV-induced thrombosis is not well-known. Several theories have been proposed, such as inhibition of p53-mediated apoptosis and platelet-derived growth factor β-receptor increase [8]. CMV infection can also cause vascular cell activation and expression of adhesion proteins, leading to increased platelet and leukocyte adhesion [7].

In our patient, the only predisposing factor one could mention is high BMI. Nevertheless, the fact that vascular thrombosis occurred during the acute phase of a CMV infection in an otherwise healthy adult with a negative thrombophilia work-up leads to a causal association between CMV infection and thrombosis. This is important, as acute mesenteric venous thrombosis carries a mortality rate of about 20% to 50% [1]. Survival depends on numerous factors, mostly on the timing of the diagnosis and surgical intervention. Another major complication of surgical treatment, which is dependent on the extent of resection, is the short-bowel syndrome [1]. In our present case, however, the patient regained normal bowel function with elimination of diarrhea by the second postoperative month.

## Conclusion

Acute CMV infection can contribute to the occurrence of mesenteric venous thrombosis in immunocompetent patients. It is important for physicians and internists to be aware of the possible thrombotic complications of CMV infection. A high level of clinical suspicion is essential to successfully treat a potentially lethal condition such as superior mesenteric venous thrombosis.

## Consent

Written informed consent was obtained from the patient for publication of this case report and any accompanying images. A copy of the written consent is available for review by the Editor-in-Chief of this journal.

## Competing interests

The authors declare that they have no competing interests.

## Authors' contributions

JK was responsible for the conception and writing of the manuscript. PB, CM and AH were responsible for drafting the manuscript. EK interpreted the computed tomographic scans. ML edited and submitted the revised manuscript. SR critically revised the manuscript and gave final approval of the version to be published. All authors read and approved the final manuscript.
